# Nurses Taking on Readiness Measures (N-TORM): A nurse-facilitated household emergency preparedness intervention feasibility study

**DOI:** 10.24298/hedn.2022-0009

**Published:** 2023-07-13

**Authors:** Tara HEAGELE, William Ellery SAMUELS, Melissa WHOLEBEN, Natasha NURSE-CLARKE, Lavonne M. ADAMS, Charleen MCNEILL

**Affiliations:** 1 Hunter-Bellevue School of Nursing, Hunter College, The City University of New York, New York, United States; 2 College of Nursing, University of Texas at El Paso, El Paso, Texas, United States; 3 School of Health Sciences, Human Services and Nursing, Herbert H. Lehman College, The City University of New York, The Bronx, New York, United States; 4 Harris College of Nursing & Health Sciences, Texas Christian University, Fort Worth, Texas, United States; 5 College of Nursing, The University of Tennessee Health Science Center, Memphis, Tennessee, United States

**Keywords:** disaster planning, disasters, emergency preparedness, intervention study, vulnerable populations

## Abstract

**Aim:**

This research evaluated the effect of a nurse-facilitated intervention on elderly or medically frail community members’ level of household emergency preparedness as measured in knowledge, actions taken, and supplies gathered. These community members had access and functional needs that must be accommodated during disasters to mitigate their increased risk of injury, illness, and death because of the disaster. With adequate preparedness, it is plausible these community members may survive the aftermath of a disaster without needing assistance from disaster responders.

**Methods:**

This was a non-randomized, single group, before-after feasibility study (*N* = 31) conducted in a one-on-one session with a nurse interventionist in an urban community setting in the United States of America. We used the Household Emergency Preparedness Instrument to measure intervention effectiveness and a Participant Experience Survey to evaluate participant satisfaction with the intervention. The intervention included an educational booklet that provided instruction to participants on how to create a disaster-related evacuation and communication plan and identify community resources. Upon completion of the booklet, participants received a complimentary disaster supply kit.

**Results:**

Mean general preparedness scores increased from 5.5 (*SD* = 4.1) pre-intervention to 20.2 (SD = 3.1) post-intervention (*p* < .001). Preparedness in all sub-scales also increased significantly (all *p*s < .001).

**Conclusions:**

Study findings provide support for the feasibility of the intervention to increase all measured aspects of emergency preparedness (knowledge, behaviors, and supplies) among elderly and medically frail adults with access and functional needs during disasters.

## INTRODUCTION

More frequent and intense extreme weather events generating widespread damage and harm to ecosystems, economies, and the environment illustrate the major impact of human-induced climate change on the world (Dinda, 2016; Howard & Sterner, 2017; Uitto et al., 2017) and place vulnerable populations at disproportionate risk for displacement, morbidity, and mortality (Heagele & Pacquiao, 2018; Pörtner et al., 2022). Disaster preparedness research over the past 20 years reveals that worldwide, households generally remain unprepared for disasters (Amberson et al., 2021). Vulnerable populations include a variety of individuals such as the elderly, the very young, pregnant people, those with complex chronic health conditions, and those with mobility, sensory, or cognitive challenges (Heagele & Pacquiao, 2018; Pörtner et al., 2022). With increased incidence of events such as severe storms, flooding, heat, and wildfires, it is crucial for healthcare workers and stakeholders to pursue interventions that support disaster risk reduction, particularly for those who are most vulnerable.

Being prepared for the conditions that disasters create, such as loss of power and potable water, or the inability to obtain supplies for a few days, may mitigate some of the hardships that disasters create. Due to social and physical barriers to adequate household emergency preparedness, community members with chronic illnesses, advanced age, and disabilities experience disproportionate disaster-related morbidity and mortality compared to other residents living in the same affected area (Bell et al., 2021). Social vulnerabilities to adequate preparedness include low socioeconomic status, low education level, language barriers, reliance on others for assistance with activities of daily living, lack of social support, and living in a high-risk geographical location (Heagele & Pacquiao, 2018).

Individuals who have complex medical conditions, physical or mental disabilities, or pharmacological dependence may be defined as “medically frail.” The United States (US) Federal Emergency Management Agency (FEMA) describes these residents as having “access and functional needs” for which accommodations must be considered during disasters in order to reduce their increased risk of injury, illness, and death as a result of the disaster (FEMA Emergency Management Institute, 2014). However, the effectiveness of preparedness for decreasing disaster-related morbidity and mortality remains unknown (Amberson et al., 2021).

Heagele (2021) conducted a qualitative study of 33 elderly and medically frail Hurricane Sandy survivors to investigate why community members did or did not prepare for disasters. Only five participants were found to have adequate preparedness, all of whom were attendees of a complimentary community intervention offered by emergency management personnel (Golden, 2013). The intervention was named Seniors Taking on Readiness Measures (STORM) and consisted of a two-part, in-person, group class offered in senior centers and community rooms of senior living communities (Golden, 2013). During the first session, participants learned about evacuation and communication planning, community resources, and the contents of a disaster supply kit. Homework prior to the second session included documenting their evacuation and communication plans. Participants who completed their homework received a free disaster supply kit during the second session. Based on the premise that the STORM intervention showed promise for improving preparedness and could be adapted for use in any setting, Heagele subsequently designed a nurse-facilitated household emergency preparedness intervention named Nurses Taking on Readiness Measures (N-TORM). The N-TORM intervention is not designed as a response or relief intervention for disaster casualties; it is a preparedness/mitigation intervention.

Heagele and Nurse-Clarke (2022) initially studied the feasibility of the N-TORM intervention with maternal/child nurses and parents of newborn infants in a large urban medical center. The intervention consisted of a one-on-one encounter with a staff nurse and at least one member of the household during the infant’s hospital stay, thus targeting caregivers of an individual (the infant) who would be vulnerable to disaster impact. Participants were provided with a community-specific preparedness booklet (NYC Emergency Management, 2022) to assist them with developing their disaster-related evacuation and communication plans; learning about local community resources to utilize before, during, and after disasters; and, understanding what is needed in the home in order to be considered prepared for a disaster. Participants who successfully completed their evacuation and family communication preparations were given a free disaster supply kit. Heagele and Nurse-Clarke reported that preparedness improved significantly after participation and that the intervention was indeed feasible among nurses and patients in a hospital setting. Given these promising initial findings, the current study sought to evaluate the effectiveness of the N-TORM intervention in other vulnerable populations more rigorously.

### Objective

The purpose of this study was to evaluate the effect of the N-TORM intervention on elderly or medically frail community members’ household emergency preparedness level as measured in knowledge, actions taken, and supplies gathered in preparation for surviving without assistance in the aftermath of a disaster. This study also served as a second test of the psychometric properties of a new preparedness instrument (Heagele et al., 2022a; Heagele et al., 2022b).

### Research Questions (RQ)

RQ_1_: What is the baseline level of household emergency preparedness of the participants?

RQ_2_: Is there a significant difference in household emergency preparedness level after receiving the N-TORM intervention?

RQ_3_: What are the perceived facilitators and barriers of nurses delivering and participants receiving the N-TORM intervention?

## METHODS

### Design

The study was a non-randomized, single group, before-after feasibility study. A theoretical framework was not used in the design or analysis of this study. The Institutional Review Board (IRB) (protocol #2022-0097-Hunter) approved the study with an “exempt with limited review” status. The participants were not compensated for their participation. Participants were able to withdraw from the study at any time with no consequences. A Cycle 51 PSC-CUNY Research Grant #63040-00 51 funded the study. The funding organization had no role in the design, implementation, interpretation, or reporting of this feasibility study.

### Participants

Recruitment and data collection began in March 2022 and concluded in May 2022. We recruited potential participants from a private Facebook group created for local residents to discuss concerns and share events happening in the county. The administrators of the group received permission to post the IRB-approved recruitment flyer. Because we experienced difficulty with finding participants willing to travel to meet an unfamiliar interventionist from a social media post, we also utilized snowball sampling. Enrolled participants could suggest other potential participants by sharing or forwarding the recruitment post. The principal investigator (PI) performed the online screening. If the potential participant screened eligible, the PI obtained informed consent and scheduled a time and place convenient to the participant to meet for the intervention.

One participant per household was eligible to participate. Inclusion criteria for the participants was being aged 18 years or older, English-speaking, medically frail (i.e., having access and functional needs during disasters) or were caregivers of individuals with access and functional needs, living independently (i.e., not in a rehabilitation facility, long-term care facility, halfway house, or prison) within one county in New Jersey, and self-identifying as feeling unprepared for disasters. Participants with cognitive impairments were excluded because they are not responsible for their preparedness activities. However, their primary caregivers were eligible to participate. Recruitment ceased at 31 successful completions of the N-TORM intervention, which was the number of disaster kits procured for this feasibility study.

### Setting

The study setting consisted of one county in New Jersey that had been heavily damaged by Hurricane Irene in 2011, Hurricane Sandy in 2012, and Hurricane Ida in 2021. This county is susceptible to flash flooding, hurricanes, thunderstorms, derechos, extreme temperatures, blizzards, ice storms, and droughts. The county is densely populated and suffered significant COVID-19 mortality, necessitating morgue trucks at the local hospitals simultaneously to New York City being the epicenter of the pandemic (The New York Times, 2022). This suggests that residents may be at risk for disproportionate morbidity and mortality during future infectious disease outbreaks compared to neighboring communities. Several households within the county are also experiencing long-term issues related to lead, legionella, and dangerous levels of chemicals in the water distributed by the local water utility (Berry, 2022). Although a preparedness intervention had been offered to adults with disabilities residing in assisted living facilities within this county, to the best of our knowledge, preparedness interventions had not been offered to independently living residents prior to this N-TORM feasibility study.

### Instrument

Measurable outcomes of this study included the intervention’s effect on the participants’ level of preparedness post-intervention as measured by the Household Emergency Preparedness Instrument (HEPI) and participant satisfaction with the intervention. Table 1 describes the different subscales of the HEPI. Participants completed the 51-question HEPI by indicating which preparedness actions they had already completed and what disaster supply kit items they had in their homes, resulting in a General Preparedness (GP) score. No evidence-based threshold GP score has yet to be established to determine what is considered prepared versus unprepared for disasters, however, higher scores indicate better preparedness. The HEPI consists of four subscales, with the Preparedness Actions/Planning (PAP) and Disaster Supplies/Resources (DSR) subscales together making up the GP score, which is applicable to all respondents. Each question comprising the GP score is scored dichotomously, and scores range from 0 to 41. On the DSR subscale, an additional point is added for each disaster supply kit item that is stored intentionally in a kit instead of coincidentally placed in the home, measuring purposeful versus incidental preparedness. Two additional subscales, Special Actions (SA) and Access and Functional Needs (AFN), are available for respondents with specific life conditions, such as having children, pets, or access and functional needs in disasters. Heagele et al. (2022a; 2022b) presented initial evidence of construct, criterion, face, and content aspects of the validity of the HEPI to capture the essentials of emergency preparedness.

To allow assessment of potential moderators and additional predictors of their preparedness levels, study participants completed a demographic form including variables of interest drawn from previous preparedness research. They also completed a one-page Participant Experience Survey to determine the clarity and ease of all steps of the N-TORM intervention and perceived preparedness post-intervention. One open-ended question was provided for participants to offer qualitative feedback on the experience.

### Intervention

This study investigated an iteration of the N-TORM intervention in which a nurse interventionist met with elderly or medically frail community members one-on-one for a single session in a community setting. At the meeting, the nurse administered the demographic data form and baseline HEPI to the participant, performed the N-TORM intervention, and then immediately readministered the HEPI along with the Participant Experience Survey.

The PI served as the nurse interventionist for this feasibility study. She is a co-creator of the N-TORM intervention and trained the trainer of the N-TORM nurse interventionists for the inpatient feasibility study mentioned earlier. Her experience includes 16 years of teaching pre- and post-licensure nursing, interdisciplinary healthcare, and community audiences.

The N-TORM intervention is designed to enable households to meet FEMA recommendations for preparedness (Ready, 2022), to safely shelter in place, and evacuate quickly with their supplies if needed. Prior to tailoring the N-TORM intervention to this study setting, the PI queried local utility companies about priority restoration registries, and police and fire departments about evacuation assistance and shelter locations. This information was gathered to ensure accurate information about these community resources could be provided to the study participants.

The N-TORM intervention consisted of educating the participants about household emergency preparedness via a *Family Disaster Plan* booklet and assisting the participants with completion of the booklet. The booklet is a hybrid of two existing community resources (New Jersey Office of Emergency Management, 2022; NYC Emergency Management, 2022). The nurse discussed the different sections of the booklet, showed the participants how to determine if their home was in flood zone, helped them register for a community emergency alert program, and offered feedback on the participants’ proposed evacuation and communication plans. The nurse instructed the participants to write their medical histories, medications, physicians, next of kin, pharmacy contact information, evacuation plan information, family communication plans, and insurance policy numbers and contact information. The booklet included preparedness recommendations, evacuation zone information, a disaster supply kit contents list, local utility company contact information, shelter locations, and special needs and priority utility restoration registry information. The participants kept their completed *Family Disaster Plan* booklets, which were not copied, collected, stored, nor used for research purposes. The booklets served as the participants’ preparedness plans and community disaster resources information source. As part of the intervention, the participants were provided with a disaster supply kit after successful completion of the booklet. The disaster supply kit was valued at $118.25 on May 10, 2023.

### ClinicalTrials.gov Identifier: NCT05455580

Hunter College of City University of New York Protocol Record 2022-0097, Nurses Taking On Readiness Measures - Mercer County, New Jersey, is registered and posted on the ClinicalTrials.gov public website.

## ANALYSIS

In addition to descriptive, bivariate analyses, we conducted hierarchical linear regressions with time (pre-test versus post-test) nested within each participant to address the research questions and evaluate the effectiveness of the N-TORM intervention and the reliability of the HEPI. Changes in level of preparedness were also evaluated through analyses of the pre- and post-intervention difference scores on the HEPI. Qualitative data obtained from the participants’ surveys were analyzed via content analysis methods.

The sample size was too small to conduct confirmatory factor analyses testing construct-related evidence of the validity of the HEPI. However, the different response scales of the HEPI subscales makes conducting a useful confirmatory factor analysis difficult even with larger sample sizes. The reliability of the HEPI was measured through internal consistency indices of the items.

## RESULTS

### Demographics

Tables 2 and 3 describe the participants’ demographic characteristics. As seen in Table 2, the mean age of the respondents was 51 years, and they reported having lived an average of 12 years in their current home and 18 years in their current community. The majority identified as female (*n* = 24, 77%), white (*n* = 27, 87%), and that they spoke English as their first language (*n* = 28, 90%). Although participants reported a variety of relationship statuses, most (*n* = 17, 55%) were married (Table 3). About two thirds (*n* = 21, 67%) were employed full time while 6 (19%) were retired. Most (*n* = 22, 71%) had completed college, either earning a baccalaureate (*n* = 4, 45%) or graduate (*n* = 8, 26%) degree.

Table 3 also shows that the participants reported rather high incomes: 23% (*n* = 7) earned $50–75,000, 42% (*n* = 13) earned $75–100,000, and 45% (*n* = 14) earned over $150,000. Not surprisingly then, 71% (*n* = 22) owned their homes, and 68% (*n* = 21) lived in detached, single houses. The composition of the respondents’ families varied but tended to be comprised of two adults between 18 and 60 years old; about one third (*n* = 11, 35%) also lived with one or two children under 18; and 29% (*n* = 9) of the households also had one or two residents over 60. Nearly all (*n* = 30, 96%) participants reported currently taking medications, less than half (n = 12, 39%) reported using medical equipment, and 4 (13%) reported being disabled. Two (6%) had served in the military.

### Reliability of HEPI Scores

The Cronbach’s *α* scores for the HEPI total and subscale scores are presented in Table 4. These measures of internal consistency ranged from .43 (for SA pre-test scores) to .82 (for DSR pre-test scores). The total GP scores displayed good reliability (*α*s = .79 & .80 at pre- and post-test, respectively) as did the PAP scores (*α*s = .73 & .77). Those of the other subscale scores varied considerably. These results suggest that concentrating on the GP scores was most advisable.

### Research Questions

#### RQ_1_: What is the baseline level of household emergency preparedness of the participants?

**Figure 1:**
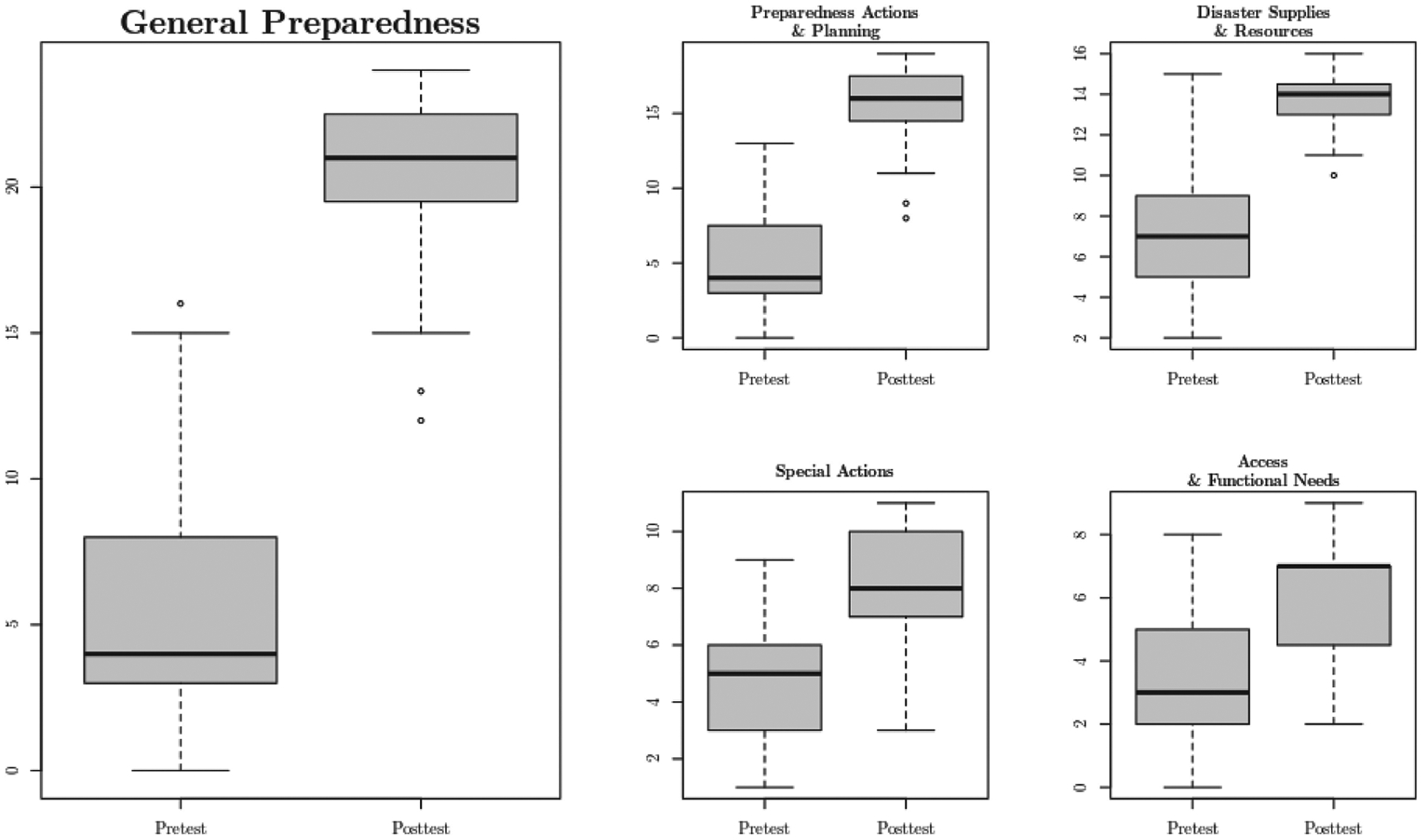
Grouped boxplot of pre- and post-survey responses by subscales.

Table 5 and Figure 1 present the mean pre- and post-test HEPI scores for all of the participants. The mean HEPI GP score at pre-test among the 31 participants was 5.5 (*SD* = 4.1). Raw GP scores could range from 0 to 41 with lower scores indicating lesser levels of preparedness. Therefore, participants objectively reported beginning the study with rather weak general preparedness.

The subscale scores in Table 5 and Figure 1 all generally show that the participants were not well prepared at pre-test, with all pre-test scores near the bottom of the respective ranges. The scores lowest to the bottom of their ranges were DSR and SA.

#### RQ_2_: Is there a significant difference in household emergency preparedness level after receiving the N-TORM intervention?

Mean GP scores increased from 5.5 (*SD* = 4.1) to 20.2 (SD = 3.1) out of 41 from pre- to post-intervention (*p* < .001). Table 6 presents the correlations between the HEPI total and subscale scores at and between pre- and post-test. All subscales except SA correlated significantly with the total GP score at pre-test; at post-test all of the subscales correlated significantly with the total score, including SA. Among the subscales, SA and AFN scores correlated well with each other at both pre- and post-test. The AFN scores also correlated significantly with the other subscales at pre-test, but only with SA at post-test.

In general, there were few significant correlations of pre-test scores with post-test scores; the exception was SA, where pre-test scores correlated significantly with post-test scores in PAP and AFN scores (as well as with their own post-test scores). It therefore appears that we cannot draw a simple picture about how pre-test levels of preparedness affected post-test levels—except, to some extent, with SA. In addition, the total GP scores tended to track the various subscales except SA. Finally, only pre-test scores in SA and AFN scores predicted their own post-test scores.

Several of the changes in HEPI scores correlated. As we can see in Table 7, all changes in subscale scores except SA correlated significantly with the overall GP score. Among the subscales, however, only changes in two pairs of subscales correlated significantly: between PAP and DSR and between PAP and AFN. Paired *t*-tests of the HEPI scores found that all significantly improved from pre- to post-test (smallest *t*_AFN Subscore_ = 6, *df*s = 30, all *p*s < .001).

#### RQ_3_: What are the perceived facilitators and barriers of nurses delivering and participants receiving the N-TORM intervention?

Seeing that the HEPI scores changed after participating in the N-TORM intervention, but also seeing that some demographic factors correlated with pre-post differences in these scores, we conducted a series of hierarchical linear regressions, nesting time (pre–post-test) within each participant. The results of these tests are presented in Table 8. This table repeats the significant pre–post differences in all HEPI scores. Gender, employment status, and family composition significantly predicted changes in the HEPI scores. However, none of the other variables—education, income, using medical equipment, and having a disability—were significantly associated with any of the HEPI scores here.

### Qualitative Data

We provided participants with an opportunity to offer comments on their experiences after completing the HEPI and the N-TORM intervention. We analyzed the open-ended question responses with qualitative content analysis. Feedback was encouraging and in support of the intervention; some recommendations were also offered (Table 9).

The average length of time it took for the nurse interventionist to perform the N-TORM intervention was 20 minutes, not including travel time to get to the participants’ locations. Participants indicated that facilitators to successful completion of the N-TORM intervention included the short time frame needed to participate, the booklet providing sufficient details to overcome their knowledge deficits, and receipt of the disaster supply kit to overcome the lack of means and time barriers to obtaining disaster supplies. The nurse encountered just two barriers to providing the N-TORM intervention: lack of time for participants to implement what they learned and the weight of the disaster supply kit. Because the intervention and follow up data collection took place in the same session, the participants did not have adequate time to complete some of the preparedness actions on the HEPI if they were not completed as of the baseline assessment. The actions that needed time to complete included discussing and drilling the disaster plan with other members of the household, taking first aid training, and installing smoke detectors.

Some participants noted that the weight of the disaster supply kit (approximately 23 pounds or 10.4 kilograms) was too heavy for them to carry on their own. This barrier can by mitigated with a bucket dolly (i.e., a cart with wheels) so that the kit can be pulled or pushed rather than carried.

Four HEPI recommendations emerged from the qualitative data. First, there were suggested improvements on the administration of the HEPI. This was the first time the HEPI was used in a paper and pencil format. Previously, the HEPI was used as an online survey only. Originally, column headings were not included on each page of the paper and pencil version, resulting in the participants having to flip between pages to determine which box they should check for their responses. Several participants asked that column headings be added to each page of the HEPI.

Second, participants who lived alone underscored the word “family” in HEPI questions 1, 6, 9, and 14, emphasizing that asking the question in this manner did not feel inclusive of people who live alone.

Third, participants felt that question 10, “In the event of an evacuation, have you considered safe and unsafe places in your community?,” and question 12, “Have you planned where to go if you had to evacuate from your home?” were redundant.

Fourth, participants were confused by question 11, “Do you know if your home is in an evacuation zone?” The intention of the question was to determine if participants live in a *known* evacuation zone, such as in a flood zone or near a nuclear power plant. Participants inquired about how they would know in advance if they were in an evacuation zone for an unplanned hazard, such as a chemical spill or wildfire.

## DISCUSSION

Qualitative feedback on the HEPI resulted in the following revisions: a) column headings were added to each page; b) the word “family” was omitted or replaced in the instrument instructions and in questions 1, 6, 9, and 14 to make the HEPI more inclusive for respondents who live alone; c) questions 10 and 12 were combined, and; d) the qualifier “(for example, in a flood zone or near a nuclear power plant)” was added to question 11 to make it clear to the respondent that we are asking if the home is in a designated evacuation zone due to a *known* hazard. After the revisions, the HEPI’s Flesch-Kincaid grade level increased slightly from 6.3 to 6.5. Even with the increase, the Flesch-Kincaid reading ease score is a 72.5, which is considered “fairly easy” to read (Moraine Park Technical College, 2022). One participant asked for a copy of the HEPI for future reference, even though she had her completed *Family Disaster Plan* booklet to keep. We received similar requests in the original pilot test of the HEPI (Heagele et al., 2022a), lending support to the possibility that the HEPI could serve as a stand-alone educational intervention.

Although this research did not claim to present a therapeutic benefit to the participants, those who successfully completed the intervention now may be self-sufficient for up to 72-hours post-disaster. The association between preparedness level and disaster resilience has not, however, been empirically tested (Heagele, 2016). In a systematic review of disaster preparedness intervention effectiveness, Amberson et al. (2021) reported that social support, educational, and behavioral interventions increased preparedness levels as measured by households completing actions related to evacuation plans, communication plans, and assembling disaster supply kits. The strength of the evidence, however, was weak. Our data adds to the body of evidence on preparedness intervention effectiveness for increasing preparedness levels of households.

This study demonstrated the feasibility of the nurse-delivered N-TORM intervention to increase all aspects of preparedness (knowledge, behaviors, and supplies) measured by the HEPI among adults with access and functional needs during disasters. These findings are important because, although preparedness of all people is critical, community members with chronic illness, advanced age, and disabilities are at increased risk of morbidity and mortality after a disaster (Bell et al., 2021; Heagele & Pacquiao, 2018). This lack of equity after a disaster is contrary to efforts to ensure the absence of avoidable disparities among socioeconomic and demographic groups or geographical areas at any time, including after a disaster (Health Resources & Services Administration, 2020; National Academies of Sciences, Engineering, and Medicine, 2021). Past research findings have demonstrated that people experience a significant increase of preparedness levels when educated about disaster preparedness by a health care provider (Al-Rousan et al., 2014; Heagele & Nurse-Clarke, 2022; Killian et al., 2017; McNeill et al., 2018). Effective nurse-delivered preparedness interventions are essential to develop and refine since nurses are the largest proportion of the health care workforce and among the most likely health care providers to spend time with patients delivering patient education (National Academies of Sciences, Engineering, and Medicine, 2021). Our research findings demonstrate that nurses can provide a quick, easy, and effective method of preparedness education to all populations - inclusive of access and functional needs, chronic diseases, or other nuances of individual and family circumstances that should be considered when crafting emergency preparedness plans and kits.

### Strengths and Limitations

This was a feasibility study and retains the limitations expected of this study design. We were unable to track the response rate for recruitment, as the recruitment flyer was posted to social media. However, we used strict inclusion and exclusion criteria, a screening and recruitment script, uniform data collection materials, and provided the intervention in the same manner to all participants to control confounders and to minimize threats to internal validity. Nevertheless, this study was at risk for selection bias related to participants’ self-enrollment and the snowball sampling. No blinding occurred. The PI served as the nurse interventionist. Social desirability bias was a risk to the internal validity of this study yet was minimized by having the participants report their level of preparedness via the paper and pencil instrument without identifiers instead of reporting it verbally to the interventionist. The baseline HEPI assessment relied on self-reported data of the participants. We did establish temporality by determining if the participants were unprepared for disasters before the intervention and prepared for disasters after the intervention. Without a contemporaneous control group, we cannot be certain that the post-test increase was due to retaking the same assessment or due to the intervention. However, the follow-up HEPI assessment was not based on self-reported data; the interventionist could objectively view the participant’s plans (upon completion of the booklet) and supplies (which were received as part of the intervention).

In this study, the N-TORM intervention educational booklet (created from locally sourced, publicly available educational resources) and the provided commercial disaster kit did not match the HEPI exactly, making the study at risk for instrumentation bias. Specifically, the disaster kit items included on the HEPI but not included in the commercial disaster kit were personal hygiene supplies like hand sanitizer and moist wipes, cash, batteries, and a fire extinguisher. Also, the kit included food and water to sustain four people for four days, whereas the HEPI asks if the respondent has enough food and water for each person in the home for one week. The booklet did not include a timeframe for food and water supplies needed. In future studies, the intervention booklet and disaster kit should be tailored to match the HEPI. Finally, intervention follow up measurement with the HEPI should be long enough to allow for time for the participants to complete the actions that could not be completed in one sitting.

External validity attends to the ability to generalize findings to other contexts. This research assessed the household emergency preparedness levels of community members with access and functional needs in one geographical location. The results may differ for community members without access and functional needs and for people in different geographical locations. However, compared to the results of a systematic review of preparedness intervention studies done with healthy and chronically ill populations in different geographical locations (Amberson et al., 2021), we achieved similar findings.

### Implications for Research and Practice

This intervention should be used in longitudinal studies to determine whether there is an association between being prepared for a disaster and surviving the disaster without the need for rescue or outside assistance. For medically frail community members, it can be determined whether there is an association between being prepared for a disaster and surviving the disaster without an acute exacerbation of a chronic illness and with no change in baseline functional status. Additional research is needed to test the sustainability of the intervention with a contemporaneous comparison group.

The revised HEPI should be administered to a large enough sample size to perform factor analysis to determine if the HEPI retains its validity and reliability characteristics. Alignment between the educational intervention and how it is measured (the HEPI) should be considered. Any resources distributed (such as booklets, quick reference guides, and checklists) should be tailored or adapted to align both with the HEPI and with the target population for the intervention.

As part of community disaster risk reduction, educational interventions aimed at populations at high risk for impact from disasters are essential. Such populations include the very young or the very old, those with chronic diseases, those with access and functional needs, and caregivers who provide support for any of the above.

For such a method to be used more widely and to expand the reach of such a model, it is essential to increase the number of nurses equipped to deliver the education. Future research directions may thus include “train the trainer” approaches to increase capacity and exploration into measuring preparedness of nurses who deliver the educational intervention. There are extant educational resources to facilitate nurses’ knowledge of emergency preparedness and response measures to increase the capacity of nursing to deliver such education (Assistant Secretary for Preparedness and Response Technical Resources, Assistance Center, and Information Exchange, 2022; Jones et al., 2014; Miller et al., 2014; National Center for Disaster Medicine and Public Health, n.d.).

Research supports the assertion that preparedness of populations significantly improves after receiving preparedness education from a healthcare provider, to include nurses (Al-Rousan et al., 2014; Heagele & Nurse-Clarke, 2022; Killian et al., 2017; McNeill et al., 2018). This suggests that further research, particularly among vulnerable populations, should explore the impact of targeted education and other interventions among those at highest risk of negative outcomes after a disaster. Further, it supports greater efforts among practicing nurses to educate clients routinely about the steps they should take to prepare for disasters inclusive of any medical or access and functional needs they might have. Such efforts align with key areas for strengthening nursing in the arena of emergency preparedness/response as outlined in *The Future of Nursing 2020*–*2030: Charting a Path to Achieve Health Equity* (National Academies of Sciences, Engineering, and Medicine, 2021). Finally, to maximize the impact nurses can have on the preparedness levels of all clients, vulnerable and not, nurses must first be educated on preparedness measures themselves. Targeted education and research in this area is an opportunity for strengthening (McNeill et al., 2020).

## CONCLUSION

Because the N-TORM intervention uses an all-hazards approach, it is possible to replicate nationally and with different vulnerable populations, tailoring the educational content to local community resources. Organizations dedicated to improving the health and safety of their communities, such as the Medical Reserve Corps and Community Emergency Response Teams, should consider using the N-TORM intervention during outreach events with residents prior to a disaster event. It is conceivable that the enduring impact of the N-TORM intervention will be lives saved and resources conserved.

## Figures and Tables

**Table 1:** Household Emergency Preparedness Instrument (HEPI) subscales

**GP** – General Preparedness	31 questions Represents the basic knowledge, actions, and disaster kit items applicable to all households
**PAP** – Preparedness Actions/Planning	19 questions Part of the GP scale
**DSR** – Disaster Supplies/Resources	12 questions Part of the GP scale
**AFN** – Access and Functional Needs	9 questions Applicable only to respondents who meet at least one of the following criteria: has a disability, is 65 years of age or older, takes at least one prescription medication, is pregnant Not part of the GP scale
**SA** – Special Actions	11 questions Applicable only to respondents who meet at least one of the following criteria: has a pet/livestock, has an infant or child, wears prescription glasses or contacts Not part of the GP scale

*Note:* (Heagele et al., 2022a; Heagele et al., 2022b).

**Table 2:** Mean age and years in current home and community

Variable	Mean	*SD*
Age	50.6	13.56
Years in Home	12.3	14.2
Years in Community	18.1	17.2

*Note*. SD = standard deviation.

**Table 3:** Demographics categories with pre- to post-test change in HEPI general preparedness scores

Variable	Value	*N*	%	GP Pre – Post Difference
Relationship	Married	17	54.8	14
	Widowed	1	3.2	20
	Divorced	5	16.1	14
	DP or Civil Union	1	3.2	9
	Single w/ Sig. Other	3	9.7	18
	Single, Never Married	4	12.9	18
Employment	Full-time	21	67.7	15
	Not employed	3	9.7	12
	Retired	6	19.4	14
	Disabled	1	3.2	15
Education	HS Diploma or GED	3	9.7	16
	Some college	4	12.9	15
	Associate degree	2	6.5	6
	Baccalaureate degree	14	45.2	15
	Graduate degree	8	25.8	15
Income	< $20,000	2	6.5	15.5
	$20–34,999	1	3.2	8
	$35–49,999	1	3.2	19
	$50–74,999	7	22.6	16.3
	$75–99,999	6	19.4	15.2
	$100–149,999	9	29.0	16.4
	$150,000 or more	5	16.1	8.8
Home Ownership	Own	22	71.0	14
	Rent	8	25.8	16
	Other	1	3.2	8
Structure	Detached Single	21	67.7	14
	Multi, 1–2 Stories	7	22.6	18
	Multi, 3 or More Stories	1	3.2	16
	Mobile or Manufactured	1	3.2	11
	No Response	1	3.2	4
Race/Ethnicity	American Native	1	3.2	15
	Asian	1	3.2	15
	Black	2	6.5	15
	Latin	2	6.5	15
	White	27	87.0	18
Uses medications	Yes	30	96.8	15
	No	1	3.2	14
Uses medical equipment	Yes	12	39.0	15
	No	19	61.0	15
Has a disability	Yes	4	13.0	16
	No	27	87.0	14
Served in military	Yes	2	6.5	12
	No	29	93.5	15
First language is English	Yes	28	90.3	15
	No	3	9.7	14
Number of residents under 18 years	0	19	61.3	15
	1	3	9.7	16
	2	8	25.8	14
	No Response	1	3.2	4
Number of residents *between* 18 and 60 years	0	5	16. 1	17
	1	7	22. 6	17
	2	14	45. 2	13
	3	2	6. 5	18
	4	1	3. 2	18
	5	1	3. 2	11
	No Response	1	3. 2	4
Number of residents over 60 years	0	21	67.7	14
	1	5	16.1	18
	2	4	12.9	15
	No Response	1	3.2	4

*Note*. DP = domestic partner; HEPI = Household Emergency Preparedness Instrument; HS = high school; GED = general education development; GP = general preparedness.

**Table 4:** Cronbach’s *α*s for the HEPI scores at pre-test and post-test

HEPI Score	Pre-test	Post-test
General Preparedness	.79	.80
Preparedness Actions & Planning	.73	.77
Disaster Supplies & Resources	.82	.27
Special Actions	.43	.47
Access & Functional Needs	.52	.71

*Note*. HEPI = Household Emergency Preparedness Instrument.

**Table 5:** Pre- and post-test HEPI total and subscale raw scores (*N* = 31)

		Pretest		Posttest	
HEPI Score	Score Range	Mean	*SD*	Mean	*SD*
General Preparedness	0–31	5.5	4.1	20.2	3.1
Preparedness Actions & Planning	0–19	5.1	3.3	15.6	2.8
Disaster Supplies & Resources	0–22	7.4	3.0	13.5	1.4
Special Actions	0–17	4.9	2.0	8.1	1.9
Access & Functional Needs	0–5	3.4	2.0	6.0	2.0

*Note*. HEPI = Household Emergency Preparedness Instrument; SD = standard deviation.

**Table 6:** Correlations within and between HEPI pre-test and post-test scores

		Pre-test					Post-test				
Time	HEPI Score	GP	PAP	DSR	SA	AFN	GP	PAP	DSR	SA	AFN
Pre-test	GP		**.95**	**.51**	.27	**.53**	.08	.04	.22	.08	−.15
	PAP	**.95**		.33	.21	**.52**	.05	.03	.14	.09	−.19
	DSR	**.51**	.33		**.52**	**.44**	.11	.09	.22	.30	.26
	SA	.27	.21	**.52**		**.39**	.35	**.39**	.15	**.54**	**.40**
	AFN	**.53**	**.52**	**.44**	**.39**		.17	.13	.25	**.37**	.28
Post-test	GP	.08	.05	.11	.35	.17		**.97**	**.52**	**.54**	**.58**
	PAP	.04	.03	.09	**.39**	.13	**.97**		**.39**	**.56**	**.64**
	DSR	.22	.14	.22	.15	.25	**.52**	**.39**		.30	.12
	SA	.08	.09	.30	**.54**	**.37**	**.54**	**.56**	.30		**.56**
	AFN	−.15	−.19	.26	**.40**	.28	**.58**	**.64**	.12	**.56**	

*Note*. Bold-faced Correlations are Significant at *α*_two-tailed_ = .05.

HEPI = Household Emergency Preparedness Instrument; GP = general preparedness; PAP = preparedness actions and planning; DSR = disaster supplies and resources; SA = special actions; AFN = access and functional needs.

**Table 7:** Correlations between pre-post differences in HEPI scores

	Pre – Post Difference in:				
Pre – Post Difference in:	GP	PAP	DSR	SA	AFN
General Preparedness		**.96**	**.41**	.27	**.69**
Preparedness Actions & Planning	**.96**		.27	.19	**.74**
Disaster Supplies & Resources	**.41**	.27		.34	.10
Special Actions	.27	.19	.34		.14
Access and Functional Needs	**.69**	**.74**	.10	.14	

*Note*. Bold-faced Correlations are Significant at *α*_two-tailed_ = .05.

HEPI = Household Emergency Preparedness Instrument; GP = general preparedness; PAP = preparedness actions and planning; DSR = disaster supplies and resources; SA = special actions; AFN = access and functional needs.

**Table 8:** Summary of hierarchical analyses of effects on HEPI scores

	General Preparedness (GP)		Preparedness Actions & Planning (PAP)		Disaster Supplies & Resources (DSR)		Special Actions (SA)		Access & Functional Needs (AFN)	
	*β*	*p*	*β*	*p*	*β*	*p*	*β*	*p*	*β*	*p*
Time (Pretest – Posttest)	**1.816**	**< .001**	**1.779**	**< .001**	**1.536**	**< .001**	**1.451**	**< .001**	**1.22**	**< .001**
Gender	−**0.275**	**0.039**	−0.222	0.084	−**0.644**	**0.003**	−**1.222**	**< .001**	−**0.721**	**0.008**
Age	−0.204	0.061	−**0.260**	**0.022**	−0.013	0.930	0.200	0.180	**0.462**	**0.030**
Relationship Type, Married vs. Other Types	0.005	0.970	0.086	0.560	−0.289	0.190	−0.200	0.350	0.103	0.710
Employment, Full Time	−**0.878**	**0.004**	−**1.032**	**0.002**	−0.546	0.15	−**1.694**	**0.001**	−0.634	0.190
Employment, Not Employed	−**0.697**	**0.035**	−**0.941**	**0.008**	0.415	0.34	−0.311	0.470	0.586	0.300
Employment, Retired	−0.827	0.069	−0.326	0.440	−**1.309**	**0.050**	−**3.173**	**< .001**	−**1.74**	**0.047**
Education	−0.032	0.63	−0.038	0.560	−0.032	0.740	−0.192	0.065	−0.177	0.180
Income	0.059	0.42	0.123	0.110	0.074	0.480	0.088	0.40	0.065	0.630
Has Medications	0.040	0.870	0.029	0.910	−0.087	0.810	**1.026**	**0.014**	−0.025	0.960
Uses Medical Equipment	0.165	0.210	−0.015	0.910	**0.642**	**0.005**	**0.582**	**0.008**	**0.589**	**0.029**
Has a Disability	0.064	0.800	0.399	0.140	−0.361	0.330	0.157	0.67	0.311	0.520
Served in Military	0.026	0.920	0.209	0.410	−0.602	0.120	−**1.413**	**0.002**	−0.005	0.990
Speaks English as First Language	−0.313	0.130	−0.306	0.130	−0.047	0.860	−0.15	0.59	−0.129	0.720
Home Ownership, Own vs. Other Types	0.104	0.580	−0.032	0.860	0.467	0.100	**1.329**	**< .001**	**1.079**	**0.010**
Years in Home	0.138	0.150	0.147	0.120	0.109	0.410	−0.086	0.51	**0.402**	**0.036**
Years in Community	−0.131	0.130	−0.133	0.120	−0.165	0.180	−0.050	0.67	−**0.432**	**0.016**
House Structure, Detached Single vs. Other Types	0.318	0.075	**0.408**	**0.029**	0.191	0.430	0.240	0.32	−0.412	0.200
Number Residents under 18 years	−**0.175**	**0.033**	−0.150	0.059	−0.219	0.058	−**0.423**	**0.002**	−0.195	00.180
Number Residents 18 to 60 years	−**0.227**	**0.011**	−**0.264**	**0.005**	−0.196	0.093	−**0.328**	**0.011**	−0.229	0.130
Number Residents over 60 years	−0.167	0.240	−**0.366**	**0.020**	0.039	0.840	0.008	0.970	−0.326	0.230

*Note*. Bold-faced Beta-Weights are Significant at *α*_two-tailed_ = .05.

**Table 9:** Participant comments about the N-TORM intervention

The process was simple and I am happy to have been given the opportunity.
This information should be provided by real estate agents to new residents.
Loved the toilet feature of the disaster kit.
Great experience overall!
Time between the two surveys would be beneficial. Wheels for [disaster kit].
What type of generator to buy? When to fill bathtubs with water? May I have a copy of the HEPI?
Experience was informative and positive. The information provided was laid out in thoughtful and concise manner.
Everything was fairly straight forward and explained clearly.
This is FANTASTIC!
Very helpful. Thanks. Learned a lot.

*Note*. N-TORM = Nurses Taking on Readiness Measures.
